# [6-(4-Bromo­phen­yl)-2,2′-bipyridine-κ^2^ *N*,*N*′](tricyclo­hexyl­phosphine-κ*P*)copper(I) tetra­fluoridoborate

**DOI:** 10.1107/S1600536810002965

**Published:** 2010-01-30

**Authors:** Xi-Long Peng

**Affiliations:** a School of Environmental and Chemical Engineering, Nanchang University, Nanchang 330031, People’s Republic of China

## Abstract

In the title compound, [Cu(C_16_H_11_BrN_2_)(C_18_H_33_P)]BF_4_, the Cu^I^ atom is three-coordinated in a distorted trigonal configuration by two N atoms from the 6-(4-bromo­phen­yl)-2,2′-bipyridine ligand and a P atom from the tricyclo­hexyl­phosphine ligand. In addition, a weak anion⋯Cu^I^ inter­action with a nearest F⋯Cu separation of 2.696 (5) Å is found.

## Related literature

For the rich photophysical properties of opper(I) complexes with diimine and phosphine ligands and their potential applications in organic light-emitting diodes (OLEDs), see: Miller *et al.* (1999 ▶); Zhang *et al.* (2006 ▶). For related structures, see: Wang *et al.* (2010 ▶). For a similar weak anion⋯Cu(I) inter­action, see: Mao *et al.* (2003 ▶).
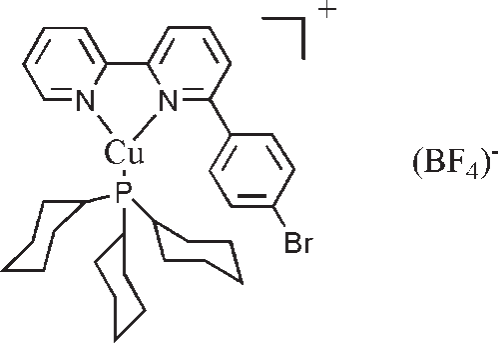


## Experimental

### 

#### Crystal data

[Cu(C_16_H_11_BrN_2_)(C_18_H_33_P)]BF_4_
*M* *_r_* = 741.94
Monoclinic, 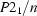

*a* = 9.8950 (8) Å
*b* = 20.2114 (17) Å
*c* = 17.3317 (14) Å
β = 93.010 (1)°
*V* = 3461.4 (5) Å^3^
*Z* = 4
Mo *K*α radiation
μ = 1.88 mm^−1^
*T* = 293 K
0.45 × 0.30 × 0.20 mm

#### Data collection

Bruker SMART CCD area-detector diffractometer
Absorption correction: multi-scan (*SADABS*; Bruker, 2001 ▶) *T*_min_ = 0.514, *T*_max_ = 0.687
26011 measured reflections
8150 independent reflections
4532 reflections with *I* > 2σ(*I*)
*R*_int_ = 0.043

#### Refinement

*R*[*F* ^2^ > 2σ(*F* ^2^)] = 0.051
*wR*(*F* ^2^) = 0.151
*S* = 1.02
8150 reflections
398 parameters
H-atom parameters constrained
Δρ_max_ = 0.36 e Å^−3^
Δρ_min_ = −0.70 e Å^−3^

### 

Data collection: *SMART* (Bruker, 1998 ▶); cell refinement: *SAINT* (Bruker, 1998 ▶); data reduction: *SAINT*; program(s) used to solve structure: *SHELXS97* (Sheldrick, 2008 ▶); program(s) used to refine structure: *SHELXL97* (Sheldrick, 2008 ▶); molecular graphics: *SHELXTL* (Sheldrick, 2008 ▶); software used to prepare material for publication: *SHELXTL*.

## Supplementary Material

Crystal structure: contains datablocks global, I. DOI: 10.1107/S1600536810002965/hg2631sup1.cif

Structure factors: contains datablocks I. DOI: 10.1107/S1600536810002965/hg2631Isup2.hkl

Additional supplementary materials:  crystallographic information; 3D view; checkCIF report

## Figures and Tables

**Fig. 1.**
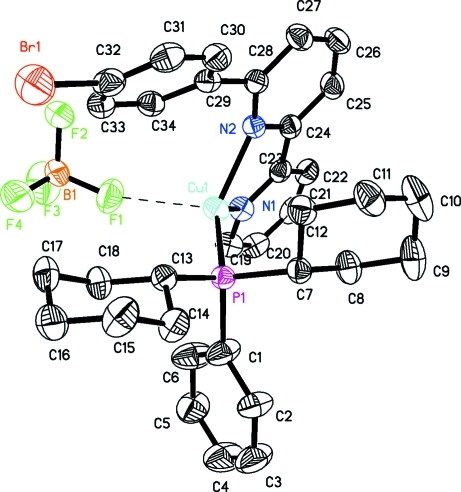
: The molecular structure of compound (I). Displacement ellipsoids are drawn at the 30% probability level, and all hydrogen atoms are omitted for clarity.

## supplementary crystallographic information

### Comment

Copper(I) complexes with diimine and phosphine ligands have attracted much attention for their rich photophysical properties and potential applications in organic light-emitting diodes (OLEDs) (Miller *et al.*, 1999; Zhang *et al.*, 2006). These complexes are generally four-coordinate. With bulky phosphine ligands such as tricyclohexylphosphine, three-coordinate complexes have been reported (Wang *et al.*, 2010). We reported here a new three-coordinated copper(I) complex of the title compound, (I).

Compound (I)

The crystal structure of (I) is depicted in Fig. 1. The copper(I) atom is three-coordinated in distorted trigonal configurations by two N atoms from 6-(4-bromo)phenyl-2,2'-bipyridine and a P atom from tricyclohexylphosphine. The coordination angles around the copper(I) atom are 80.029 (11) ° (N1—Cu1—N2), 131.74 (8) ° (N1—Cu1—P1) and 129.43 (7) ° (P1—Cu1—N2) respectively. The Cu—P (2.1811 (9) Å) and Cu—N (2.038 (3) and 2.080 (3) Å) distances are within the normal ranges for related complexes (Wang *et al.*, 2010). In addition, weak anion···Cu(I) interaction is founded, as evidenced by the nearest F···Cu separation of 2.696 (5) Å (Cu1—F1) in the title compound. Similar weak anion···Cu(I) interaction was also reported by Mao *et al.* (2003).

### Experimental

The ligand 6-(4-bromo)phenyl-2,2'-bipyridine (L) was prepared by literature method (Wang *et al.*, 2010). A mixture of [Cu(CH_3_CN)_4_]BF_4_ (100 mg, 0.32 mmol) and L (99 mg, 0.32 mmol) in dichloromethane (20 ml) was stirred under nitrogen atmosphere at room temperature for 2 h. Then tricyclohexylphosphine (89 mg, 0.32 mmol) was added kept stirring for 2 h. The solvents were removed and the solid residue was afforded. Yellow single crystals suitable for X-ray diffraction were obtained from the solution of dichloromethane by vapor diffusion with diethyl ether (yield: 82%). Analysis calculated for [Cu(C_16_H_11_N_2_Br)(C_18_H_33_P)].(BF_4_): C 53.38, H 5.93 N 3.77%; Found: C 53.92, H 5.63, 3.57%.

### Refinement

All H atoms were positioned geomertrically and treated as riding (C—H = 0.97 Å for cyclohexyl and C—H = 0.93 Å otherwise) with U_iso_(H) = 1.2 U_eq_(C) of the carrier atom.

### Crystal data

**Table:**

[Cu(C_16_H_11_BrN_2_)(C_18_H_33_P)]BF_4_	*F*(000) = 1528
*M**_r_* = 741.94	*D*_x_ = 1.424 Mg m^−^^3^
Monoclinic, *P*2_1_/*n*	Mo *K*α radiation, λ = 0.71073 Å
Hall symbol: -P 2yn	Cell parameters from 892 reflections
*a* = 9.8950 (8) Å	θ = 2.2–25.8°
*b* = 20.2114 (17) Å	µ = 1.88 mm^−^^1^
*c* = 17.3317 (14) Å	*T* = 293 K
β = 93.010 (1)°	Block, yellow
*V* = 3461.4 (5) Å^3^	0.45 × 0.30 × 0.20 mm
*Z* = 4	

### Data collection

**Table:**

Bruker SMART CCD area-detector diffractometer	8150 independent reflections
Radiation source: fine-focus sealed tube	4532 reflections with *I* > 2σ(*I*)
graphite	*R*_int_ = 0.043
phi and ω scans	θ_max_ = 28.3°, θ_min_ = 2.0°
Absorption correction: multi-scan (*SADABS*; Bruker, 2001)	*h* = −13→13
*T*_min_ = 0.514, *T*_max_ = 0.687	*k* = −26→26
26011 measured reflections	*l* = −23→21

### Refinement

**Table:**

Refinement on *F*^2^	Primary atom site location: structure-invariant direct methods
Least-squares matrix: full	Secondary atom site location: difference Fourier map
*R*[*F*^2^ > 2σ(*F*^2^)] = 0.051	Hydrogen site location: inferred from neighbouring sites
*wR*(*F*^2^) = 0.151	H-atom parameters constrained
*S* = 1.02	*w* = 1/[σ^2^(*F*_o_^2^) + (0.059*P*)^2^ + 1.0457*P*] where *P* = (*F*_o_^2^ + 2*F*_c_^2^)/3
8150 reflections	(Δ/σ)_max_ = 0.012
398 parameters	Δρ_max_ = 0.36 e Å^−^^3^
0 restraints	Δρ_min_ = −0.70 e Å^−^^3^

### Special details

**Table:**

Geometry. All esds (except the esd in the dihedral angle between two l.s. planes) are estimated using the full covariance matrix. The cell esds are taken into account individually in the estimation of esds in distances, angles and torsion angles; correlations between esds in cell parameters are only used when they are defined by crystal symmetry. An approximate (isotropic) treatment of cell esds is used for estimating esds involving l.s. planes.
Refinement. Refinement of *F*^2^ against ALL reflections. The weighted *R*-factor wR and goodness of fit *S* are based on *F*^2^, conventional *R*-factors *R* are based on *F*, with *F* set to zero for negative *F*^2^. The threshold expression of *F*^2^ > σ(*F*^2^) is used only for calculating *R*-factors(gt) etc. and is not relevant to the choice of reflections for refinement. *R*-factors based on *F*^2^ are statistically about twice as large as those based on *F*, and *R*- factors based on ALL data will be even larger.

### Fractional atomic coordinates and isotropic or equivalent isotropic displacement parameters (Å^2^)

**Table:**

	*x*	*y*	*z*	*U*_iso_*/*U*_eq_	
Cu1	0.68710 (4)	0.11177 (2)	0.13751 (2)	0.05341 (15)	
P1	0.77547 (9)	0.19570 (4)	0.20113 (5)	0.0467 (2)	
Br1	0.78459 (6)	0.00217 (4)	0.49674 (3)	0.1190 (3)	
N2	0.4943 (3)	0.07131 (12)	0.14102 (15)	0.0469 (6)	
N1	0.6442 (3)	0.09700 (14)	0.02248 (16)	0.0542 (7)	
C1	0.8915 (5)	0.24251 (19)	0.1413 (3)	0.0848 (14)	
H1A	0.9664	0.2341	0.1792	0.102*	
C2	0.9125 (5)	0.3136 (2)	0.1498 (3)	0.0845 (13)	
H2A	0.9489	0.3218	0.2020	0.101*	
H2B	0.8248	0.3350	0.1446	0.101*	
C3	1.0017 (6)	0.3458 (2)	0.0961 (4)	0.117 (2)	
H3A	0.9493	0.3805	0.0700	0.140*	
H3B	1.0730	0.3675	0.1272	0.140*	
C4	1.0649 (6)	0.3095 (3)	0.0385 (3)	0.118 (2)	
H4A	1.1614	0.3176	0.0454	0.142*	
H4B	1.0355	0.3286	−0.0110	0.142*	
C5	1.0469 (5)	0.2393 (3)	0.0321 (3)	0.0971 (16)	
H5A	1.1355	0.2189	0.0382	0.117*	
H5B	1.0120	0.2298	−0.0201	0.117*	
C6	0.9594 (6)	0.2066 (2)	0.0848 (3)	0.1063 (19)	
H6A	0.8906	0.1834	0.0535	0.128*	
H6B	1.0135	0.1731	0.1121	0.128*	
C7	0.6381 (3)	0.25423 (16)	0.22294 (19)	0.0517 (8)	
H7A	0.6789	0.2952	0.2435	0.062*	
C8	0.5519 (4)	0.2706 (2)	0.1495 (2)	0.0724 (11)	
H8A	0.6081	0.2920	0.1127	0.087*	
H8B	0.5174	0.2299	0.1263	0.087*	
C13	0.8643 (3)	0.17867 (16)	0.29569 (18)	0.0515 (8)	
H13A	0.7983	0.1551	0.3257	0.062*	
C24	0.4262 (4)	0.07942 (15)	0.07240 (19)	0.0509 (8)	
C28	0.4265 (3)	0.05544 (15)	0.2040 (2)	0.0510 (8)	
C29	0.5105 (4)	0.04185 (16)	0.27528 (19)	0.0510 (8)	
C23	0.5110 (4)	0.09002 (15)	0.00546 (19)	0.0517 (8)	
C33	0.7185 (4)	0.00015 (19)	0.3355 (2)	0.0668 (10)	
H33A	0.8025	−0.0200	0.3318	0.080*	
F2	0.8170 (3)	−0.09236 (13)	0.17853 (19)	0.1044 (9)	
C19	0.7271 (4)	0.10229 (19)	−0.0355 (2)	0.0651 (10)	
H19A	0.8196	0.1055	−0.0237	0.078*	
C34	0.6373 (4)	0.01317 (17)	0.2707 (2)	0.0583 (9)	
H34A	0.6680	0.0025	0.2224	0.070*	
C22	0.4591 (4)	0.09145 (17)	−0.0705 (2)	0.0631 (10)	
H22A	0.3664	0.0878	−0.0817	0.076*	
C27	0.2865 (4)	0.05087 (18)	0.1998 (2)	0.0635 (10)	
H27A	0.2403	0.0409	0.2437	0.076*	
C32	0.6723 (5)	0.0178 (2)	0.4065 (2)	0.0713 (11)	
C12	0.5487 (4)	0.22462 (19)	0.2837 (2)	0.0649 (10)	
H12A	0.5164	0.1816	0.2663	0.078*	
H12B	0.6024	0.2184	0.3316	0.078*	
C18	0.9811 (4)	0.1305 (2)	0.2887 (2)	0.0670 (10)	
H18A	0.9492	0.0916	0.2606	0.080*	
H18B	1.0508	0.1511	0.2596	0.080*	
C14	0.9064 (5)	0.23832 (19)	0.3445 (2)	0.0720 (11)	
H14A	0.9728	0.2639	0.3181	0.086*	
H14B	0.8282	0.2663	0.3512	0.086*	
C30	0.4678 (4)	0.05694 (17)	0.3477 (2)	0.0625 (10)	
H30A	0.3826	0.0755	0.3520	0.075*	
C17	1.0409 (5)	0.1100 (2)	0.3669 (2)	0.0748 (11)	
H17A	1.1191	0.0820	0.3602	0.090*	
H17B	0.9747	0.0843	0.3934	0.090*	
C26	0.2172 (4)	0.06113 (19)	0.1308 (2)	0.0696 (11)	
H26A	0.1232	0.0588	0.1277	0.084*	
C20	0.6821 (5)	0.10323 (18)	−0.1120 (2)	0.0718 (11)	
H20A	0.7427	0.1071	−0.1510	0.086*	
F1	0.8562 (3)	0.00766 (13)	0.12988 (18)	0.1024 (9)	
B1	0.9142 (5)	−0.0521 (3)	0.1505 (3)	0.0689 (12)	
C15	0.9660 (6)	0.2171 (2)	0.4231 (2)	0.0899 (15)	
H15A	0.9973	0.2559	0.4519	0.108*	
H15B	0.8963	0.1960	0.4518	0.108*	
C31	0.5477 (5)	0.04533 (19)	0.4138 (2)	0.0697 (11)	
H31A	0.5174	0.0560	0.4621	0.084*	
C25	0.2863 (4)	0.07504 (19)	0.0657 (2)	0.0656 (10)	
H25A	0.2400	0.0813	0.0182	0.079*	
C21	0.5465 (5)	0.09833 (18)	−0.1291 (2)	0.0731 (12)	
H21A	0.5131	0.0996	−0.1803	0.088*	
F4	1.0131 (3)	−0.04289 (16)	0.20553 (19)	0.1233 (11)	
C11	0.4289 (4)	0.2684 (2)	0.2986 (3)	0.0807 (12)	
H11A	0.4609	0.3092	0.3227	0.097*	
H11B	0.3715	0.2462	0.3342	0.097*	
F3	0.9654 (4)	−0.07998 (18)	0.08765 (19)	0.1367 (12)	
C16	1.0830 (5)	0.1695 (2)	0.4158 (3)	0.0908 (14)	
H16A	1.1148	0.1548	0.4668	0.109*	
H16B	1.1569	0.1922	0.3925	0.109*	
C10	0.3467 (5)	0.2845 (3)	0.2250 (3)	0.1079 (19)	
H10A	0.3059	0.2443	0.2039	0.130*	
H10B	0.2745	0.3148	0.2365	0.130*	
C9	0.4344 (5)	0.3154 (3)	0.1659 (3)	0.0993 (16)	
H9A	0.4686	0.3576	0.1850	0.119*	
H9B	0.3805	0.3234	0.1184	0.119*	

### Atomic displacement parameters (Å^2^)

**Table:**

	*U*^11^	*U*^22^	*U*^33^	*U*^12^	*U*^13^	*U*^23^
Cu1	0.0505 (3)	0.0598 (3)	0.0497 (3)	−0.0065 (2)	0.00034 (18)	−0.00971 (18)
P1	0.0461 (5)	0.0488 (5)	0.0448 (5)	−0.0027 (4)	−0.0008 (4)	−0.0016 (4)
Br1	0.1016 (5)	0.1800 (6)	0.0738 (4)	−0.0118 (4)	−0.0103 (3)	0.0430 (3)
N2	0.0463 (16)	0.0445 (14)	0.0495 (16)	−0.0020 (12)	−0.0010 (13)	−0.0071 (11)
N1	0.0588 (19)	0.0575 (17)	0.0464 (15)	−0.0013 (14)	0.0040 (14)	−0.0103 (12)
C1	0.104 (4)	0.055 (2)	0.100 (3)	−0.006 (2)	0.050 (3)	0.005 (2)
C2	0.103 (4)	0.061 (2)	0.091 (3)	−0.020 (2)	0.021 (3)	0.004 (2)
C3	0.128 (5)	0.071 (3)	0.158 (6)	−0.007 (3)	0.062 (4)	0.027 (3)
C4	0.131 (5)	0.112 (4)	0.117 (4)	−0.028 (4)	0.056 (4)	0.021 (3)
C5	0.106 (4)	0.097 (4)	0.092 (3)	0.006 (3)	0.043 (3)	0.017 (3)
C6	0.131 (5)	0.083 (3)	0.111 (4)	−0.030 (3)	0.065 (4)	−0.021 (3)
C7	0.048 (2)	0.0499 (18)	0.0562 (19)	0.0000 (15)	−0.0035 (15)	−0.0059 (15)
C8	0.066 (3)	0.086 (3)	0.064 (2)	0.014 (2)	−0.009 (2)	−0.004 (2)
C13	0.053 (2)	0.0535 (19)	0.0472 (19)	−0.0032 (16)	−0.0035 (15)	−0.0024 (14)
C24	0.052 (2)	0.0411 (17)	0.058 (2)	0.0029 (15)	−0.0029 (16)	−0.0088 (14)
C28	0.051 (2)	0.0431 (18)	0.060 (2)	0.0009 (15)	0.0083 (16)	−0.0058 (15)
C29	0.053 (2)	0.0465 (18)	0.054 (2)	−0.0054 (16)	0.0124 (16)	−0.0032 (14)
C23	0.059 (2)	0.0389 (17)	0.056 (2)	−0.0003 (15)	−0.0055 (17)	−0.0051 (14)
C33	0.059 (2)	0.074 (3)	0.068 (3)	0.0010 (19)	0.008 (2)	0.0142 (19)
F2	0.0653 (16)	0.0881 (17)	0.160 (3)	−0.0037 (14)	0.0074 (17)	0.0175 (17)
C19	0.069 (3)	0.072 (2)	0.054 (2)	−0.0079 (19)	0.0067 (19)	−0.0102 (17)
C34	0.057 (2)	0.061 (2)	0.058 (2)	−0.0032 (18)	0.0107 (18)	−0.0006 (16)
C22	0.074 (3)	0.057 (2)	0.056 (2)	0.0006 (18)	−0.0167 (19)	−0.0014 (16)
C27	0.053 (2)	0.066 (2)	0.073 (3)	0.0020 (18)	0.014 (2)	−0.0090 (18)
C32	0.078 (3)	0.078 (3)	0.059 (2)	−0.014 (2)	0.001 (2)	0.0165 (19)
C12	0.063 (2)	0.064 (2)	0.069 (2)	0.0038 (19)	0.0127 (19)	−0.0100 (18)
C18	0.063 (3)	0.078 (3)	0.059 (2)	0.012 (2)	−0.0071 (19)	0.0004 (18)
C14	0.092 (3)	0.061 (2)	0.061 (2)	0.001 (2)	−0.022 (2)	−0.0081 (18)
C30	0.065 (2)	0.061 (2)	0.063 (2)	0.0029 (18)	0.0165 (19)	−0.0004 (17)
C17	0.069 (3)	0.088 (3)	0.067 (3)	0.019 (2)	−0.006 (2)	0.009 (2)
C26	0.045 (2)	0.073 (3)	0.091 (3)	−0.0029 (19)	0.003 (2)	−0.006 (2)
C20	0.101 (4)	0.061 (2)	0.054 (2)	−0.008 (2)	0.012 (2)	−0.0045 (17)
F1	0.104 (2)	0.0866 (17)	0.118 (2)	0.0332 (15)	0.0251 (17)	0.0238 (15)
B1	0.051 (3)	0.076 (3)	0.081 (3)	0.007 (2)	0.009 (2)	0.000 (2)
C15	0.133 (4)	0.078 (3)	0.055 (2)	0.000 (3)	−0.028 (3)	−0.004 (2)
C31	0.087 (3)	0.071 (2)	0.052 (2)	−0.008 (2)	0.014 (2)	0.0030 (18)
C25	0.055 (2)	0.063 (2)	0.077 (3)	0.0020 (18)	−0.0142 (19)	−0.0041 (19)
C21	0.117 (4)	0.054 (2)	0.046 (2)	0.001 (2)	−0.012 (2)	−0.0041 (16)
F4	0.099 (2)	0.130 (2)	0.135 (3)	−0.0346 (19)	−0.0452 (19)	0.0268 (19)
C11	0.062 (3)	0.095 (3)	0.085 (3)	0.009 (2)	0.012 (2)	−0.024 (2)
F3	0.137 (3)	0.155 (3)	0.121 (3)	0.051 (2)	0.036 (2)	−0.024 (2)
C16	0.083 (3)	0.108 (4)	0.077 (3)	−0.010 (3)	−0.032 (2)	0.016 (3)
C10	0.059 (3)	0.156 (5)	0.108 (4)	0.033 (3)	−0.009 (3)	−0.044 (4)
C9	0.086 (4)	0.121 (4)	0.087 (3)	0.047 (3)	−0.027 (3)	−0.014 (3)

### Geometric parameters (Å, °)

**Table:**

Cu1—N1	2.038 (3)	F2—B1	1.368 (5)
Cu1—N2	2.080 (3)	C19—C20	1.376 (5)
Cu1—P1	2.1811 (9)	C19—H19A	0.9300
P1—C1	1.848 (4)	C34—H34A	0.9300
P1—C13	1.851 (3)	C22—C21	1.375 (6)
P1—C7	1.855 (3)	C22—H22A	0.9300
Br1—C32	1.896 (4)	C27—C26	1.362 (5)
N2—C24	1.346 (4)	C27—H27A	0.9300
N2—C28	1.349 (4)	C32—C31	1.365 (6)
N1—C19	1.335 (4)	C12—C11	1.512 (5)
N1—C23	1.342 (4)	C12—H12A	0.9700
C1—C6	1.416 (5)	C12—H12B	0.9700
C1—C2	1.457 (5)	C18—C17	1.507 (5)
C1—H1A	0.9800	C18—H18A	0.9700
C2—C3	1.468 (6)	C18—H18B	0.9700
C2—H2A	0.9700	C14—C15	1.518 (5)
C2—H2B	0.9700	C14—H14A	0.9700
C3—C4	1.412 (7)	C14—H14B	0.9700
C3—H3A	0.9700	C30—C31	1.377 (6)
C3—H3B	0.9700	C30—H30A	0.9300
C4—C5	1.434 (7)	C17—C16	1.519 (6)
C4—H4A	0.9700	C17—H17A	0.9700
C4—H4B	0.9700	C17—H17B	0.9700
C5—C6	1.451 (6)	C26—C25	1.380 (5)
C5—H5A	0.9700	C26—H26A	0.9300
C5—H5B	0.9700	C20—C21	1.362 (6)
C6—H6A	0.9700	C20—H20A	0.9300
C6—H6B	0.9700	F1—B1	1.376 (5)
C7—C12	1.532 (5)	B1—F4	1.343 (6)
C7—C8	1.531 (5)	B1—F3	1.349 (5)
C7—H7A	0.9800	C15—C16	1.515 (7)
C8—C9	1.512 (6)	C15—H15A	0.9700
C8—H8A	0.9700	C15—H15B	0.9700
C8—H8B	0.9700	C31—H31A	0.9300
C13—C14	1.519 (5)	C25—H25A	0.9300
C13—C18	1.520 (5)	C21—H21A	0.9300
C13—H13A	0.9800	C11—C10	1.512 (7)
C24—C25	1.385 (5)	C11—H11A	0.9700
C24—C23	1.483 (5)	C11—H11B	0.9700
C28—C27	1.386 (5)	C16—H16A	0.9700
C28—C29	1.479 (5)	C16—H16B	0.9700
C29—C30	1.379 (5)	C10—C9	1.513 (7)
C29—C34	1.388 (5)	C10—H10A	0.9700
C23—C22	1.389 (5)	C10—H10B	0.9700
C33—C34	1.372 (5)	C9—H9A	0.9700
C33—C32	1.382 (6)	C9—H9B	0.9700
C33—H33A	0.9300		
			
N1—Cu1—N2	80.02 (11)	C29—C34—H34A	119.2
N1—Cu1—P1	131.74 (8)	C21—C22—C23	119.1 (4)
N2—Cu1—P1	129.43 (7)	C21—C22—H22A	120.4
C1—P1—C13	108.2 (2)	C23—C22—H22A	120.4
C1—P1—C7	105.74 (18)	C26—C27—C28	119.5 (4)
C13—P1—C7	104.91 (15)	C26—C27—H27A	120.3
C1—P1—Cu1	111.03 (15)	C28—C27—H27A	120.3
C13—P1—Cu1	117.53 (11)	C31—C32—C33	122.0 (4)
C7—P1—Cu1	108.68 (11)	C31—C32—Br1	119.0 (3)
C24—N2—C28	119.8 (3)	C33—C32—Br1	119.0 (3)
C24—N2—Cu1	110.1 (2)	C11—C12—C7	112.0 (3)
C28—N2—Cu1	127.8 (2)	C11—C12—H12A	109.2
C19—N1—C23	118.5 (3)	C7—C12—H12A	109.2
C19—N1—Cu1	128.3 (3)	C11—C12—H12B	109.2
C23—N1—Cu1	112.5 (2)	C7—C12—H12B	109.2
C6—C1—C2	120.3 (4)	H12A—C12—H12B	107.9
C6—C1—P1	117.2 (3)	C17—C18—C13	111.7 (3)
C2—C1—P1	122.5 (3)	C17—C18—H18A	109.3
C6—C1—H1A	90.3	C13—C18—H18A	109.3
C2—C1—H1A	90.3	C17—C18—H18B	109.3
P1—C1—H1A	90.3	C13—C18—H18B	109.3
C1—C2—C3	117.5 (4)	H18A—C18—H18B	107.9
C1—C2—H2A	107.9	C15—C14—C13	111.0 (3)
C3—C2—H2A	107.9	C15—C14—H14A	109.4
C1—C2—H2B	107.9	C13—C14—H14A	109.4
C3—C2—H2B	107.9	C15—C14—H14B	109.4
H2A—C2—H2B	107.2	C13—C14—H14B	109.4
C4—C3—C2	121.4 (4)	H14A—C14—H14B	108.0
C4—C3—H3A	107.0	C31—C30—C29	122.0 (4)
C2—C3—H3A	107.0	C31—C30—H30A	119.0
C4—C3—H3B	107.0	C29—C30—H30A	119.0
C2—C3—H3B	107.0	C18—C17—C16	111.5 (3)
H3A—C3—H3B	106.7	C18—C17—H17A	109.3
C3—C4—C5	120.8 (4)	C16—C17—H17A	109.3
C3—C4—H4A	107.1	C18—C17—H17B	109.3
C5—C4—H4A	107.1	C16—C17—H17B	109.3
C3—C4—H4B	107.1	H17A—C17—H17B	108.0
C5—C4—H4B	107.1	C27—C26—C25	120.0 (4)
H4A—C4—H4B	106.8	C27—C26—H26A	120.0
C4—C5—C6	118.5 (4)	C25—C26—H26A	120.0
C4—C5—H5A	107.7	C21—C20—C19	118.3 (4)
C6—C5—H5A	107.7	C21—C20—H20A	120.9
C4—C5—H5B	107.7	C19—C20—H20A	120.9
C6—C5—H5B	107.7	F4—B1—F3	109.9 (4)
H5A—C5—H5B	107.1	F4—B1—F2	109.3 (4)
C1—C6—C5	121.6 (4)	F3—B1—F2	109.8 (4)
C1—C6—H6A	106.9	F4—B1—F1	110.0 (4)
C5—C6—H6A	106.9	F3—B1—F1	109.0 (4)
C1—C6—H6B	106.9	F2—B1—F1	108.8 (4)
C5—C6—H6B	106.9	C16—C15—C14	111.5 (4)
H6A—C6—H6B	106.7	C16—C15—H15A	109.3
C12—C7—C8	109.8 (3)	C14—C15—H15A	109.3
C12—C7—P1	110.3 (2)	C16—C15—H15B	109.3
C8—C7—P1	110.5 (2)	C14—C15—H15B	109.3
C12—C7—H7A	108.7	H15A—C15—H15B	108.0
C8—C7—H7A	108.7	C32—C31—C30	118.3 (4)
P1—C7—H7A	108.7	C32—C31—H31A	120.8
C9—C8—C7	111.9 (3)	C30—C31—H31A	120.8
C9—C8—H8A	109.2	C26—C25—C24	118.7 (4)
C7—C8—H8A	109.2	C26—C25—H25A	120.6
C9—C8—H8B	109.2	C24—C25—H25A	120.6
C7—C8—H8B	109.2	C20—C21—C22	119.8 (4)
H8A—C8—H8B	107.9	C20—C21—H21A	120.1
C14—C13—C18	111.4 (3)	C22—C21—H21A	120.1
C14—C13—P1	116.7 (2)	C12—C11—C10	111.9 (3)
C18—C13—P1	112.1 (2)	C12—C11—H11A	109.2
C14—C13—H13A	105.1	C10—C11—H11A	109.2
C18—C13—H13A	105.1	C12—C11—H11B	109.2
P1—C13—H13A	105.1	C10—C11—H11B	109.2
N2—C24—C25	121.2 (3)	H11A—C11—H11B	107.9
N2—C24—C23	115.5 (3)	C15—C16—C17	111.2 (4)
C25—C24—C23	123.2 (3)	C15—C16—H16A	109.4
N2—C28—C27	120.7 (3)	C17—C16—H16A	109.4
N2—C28—C29	116.0 (3)	C15—C16—H16B	109.4
C27—C28—C29	123.2 (3)	C17—C16—H16B	109.4
C30—C29—C34	117.7 (3)	H16A—C16—H16B	108.0
C30—C29—C28	122.3 (3)	C9—C10—C11	111.0 (4)
C34—C29—C28	120.0 (3)	C9—C10—H10A	109.4
N1—C23—C22	121.1 (3)	C11—C10—H10A	109.4
N1—C23—C24	115.6 (3)	C9—C10—H10B	109.4
C22—C23—C24	123.3 (3)	C11—C10—H10B	109.4
C34—C33—C32	118.2 (4)	H10A—C10—H10B	108.0
C34—C33—H33A	120.9	C10—C9—C8	110.7 (4)
C32—C33—H33A	120.9	C10—C9—H9A	109.5
N1—C19—C20	123.1 (4)	C8—C9—H9A	109.5
N1—C19—H19A	118.5	C10—C9—H9B	109.5
C20—C19—H19A	118.5	C8—C9—H9B	109.5
C33—C34—C29	121.7 (3)	H9A—C9—H9B	108.1
C33—C34—H34A	119.2		
			
N1—Cu1—P1—C1	−29.0 (2)	N2—C28—C29—C30	147.1 (3)
N2—Cu1—P1—C1	−144.6 (2)	C27—C28—C29—C30	−34.5 (5)
N1—Cu1—P1—C13	−154.23 (16)	N2—C28—C29—C34	−32.6 (4)
N2—Cu1—P1—C13	90.12 (16)	C27—C28—C29—C34	145.7 (3)
N1—Cu1—P1—C7	86.93 (16)	C19—N1—C23—C22	−3.1 (5)
N2—Cu1—P1—C7	−28.73 (15)	Cu1—N1—C23—C22	168.3 (3)
N1—Cu1—N2—C24	−22.3 (2)	C19—N1—C23—C24	175.6 (3)
P1—Cu1—N2—C24	114.66 (19)	Cu1—N1—C23—C24	−13.0 (3)
N1—Cu1—N2—C28	175.3 (3)	N2—C24—C23—N1	−6.6 (4)
P1—Cu1—N2—C28	−47.8 (3)	C25—C24—C23—N1	176.2 (3)
N2—Cu1—N1—C19	−170.5 (3)	N2—C24—C23—C22	172.1 (3)
P1—Cu1—N1—C19	54.4 (3)	C25—C24—C23—C22	−5.1 (5)
N2—Cu1—N1—C23	19.1 (2)	C23—N1—C19—C20	2.3 (5)
P1—Cu1—N1—C23	−115.9 (2)	Cu1—N1—C19—C20	−167.5 (3)
C13—P1—C1—C6	100.5 (5)	C32—C33—C34—C29	−1.5 (5)
C7—P1—C1—C6	−147.6 (4)	C30—C29—C34—C33	−0.1 (5)
Cu1—P1—C1—C6	−29.9 (5)	C28—C29—C34—C33	179.6 (3)
C13—P1—C1—C2	−80.5 (5)	N1—C23—C22—C21	1.8 (5)
C7—P1—C1—C2	31.5 (5)	C24—C23—C22—C21	−176.8 (3)
Cu1—P1—C1—C2	149.2 (4)	N2—C28—C27—C26	1.4 (5)
C6—C1—C2—C3	2.2 (8)	C29—C28—C27—C26	−176.9 (3)
P1—C1—C2—C3	−176.8 (4)	C34—C33—C32—C31	2.2 (6)
C1—C2—C3—C4	−0.1 (9)	C34—C33—C32—Br1	−178.5 (3)
C2—C3—C4—C5	−1.8 (10)	C8—C7—C12—C11	−53.4 (4)
C3—C4—C5—C6	1.6 (10)	P1—C7—C12—C11	−175.5 (3)
C2—C1—C6—C5	−2.5 (9)	C14—C13—C18—C17	54.7 (4)
P1—C1—C6—C5	176.6 (5)	P1—C13—C18—C17	−172.4 (3)
C4—C5—C6—C1	0.6 (9)	C18—C13—C14—C15	−54.7 (5)
C1—P1—C7—C12	−170.3 (3)	P1—C13—C14—C15	174.7 (3)
C13—P1—C7—C12	−56.1 (3)	C34—C29—C30—C31	1.0 (5)
Cu1—P1—C7—C12	70.4 (3)	C28—C29—C30—C31	−178.7 (3)
C1—P1—C7—C8	68.0 (3)	C13—C18—C17—C16	−54.7 (5)
C13—P1—C7—C8	−177.7 (3)	C28—C27—C26—C25	0.9 (6)
Cu1—P1—C7—C8	−51.2 (3)	N1—C19—C20—C21	−0.2 (6)
C12—C7—C8—C9	55.1 (5)	C13—C14—C15—C16	55.3 (5)
P1—C7—C8—C9	177.0 (3)	C33—C32—C31—C30	−1.4 (6)
C1—P1—C13—C14	62.4 (3)	Br1—C32—C31—C30	179.4 (3)
C7—P1—C13—C14	−50.2 (3)	C29—C30—C31—C32	−0.3 (6)
Cu1—P1—C13—C14	−171.0 (3)	C27—C26—C25—C24	−1.2 (6)
C1—P1—C13—C18	−67.9 (3)	N2—C24—C25—C26	−0.9 (5)
C7—P1—C13—C18	179.6 (3)	C23—C24—C25—C26	176.1 (3)
Cu1—P1—C13—C18	58.8 (3)	C19—C20—C21—C22	−1.2 (6)
C28—N2—C24—C25	3.3 (5)	C23—C22—C21—C20	0.4 (5)
Cu1—N2—C24—C25	−160.8 (3)	C7—C12—C11—C10	54.4 (5)
C28—N2—C24—C23	−174.0 (3)	C14—C15—C16—C17	−55.4 (5)
Cu1—N2—C24—C23	21.9 (3)	C18—C17—C16—C15	55.0 (5)
C24—N2—C28—C27	−3.5 (5)	C12—C11—C10—C9	−55.5 (6)
Cu1—N2—C28—C27	157.4 (2)	C11—C10—C9—C8	56.5 (6)
C24—N2—C28—C29	174.9 (3)	C7—C8—C9—C10	−57.2 (5)
Cu1—N2—C28—C29	−24.2 (4)		
